# Effectiveness of health literacy interventions on anxious and depressive symptomatology in primary health care: A systematic review and meta-analysis

**DOI:** 10.3389/fpubh.2023.1007238

**Published:** 2023-02-09

**Authors:** Rosa Magallón-Botaya, Fátima Méndez-López, Bárbara Oliván-Blázquez, Luis Carlos Silva-Aycaguer, David Lerma-Irureta, Cruz Bartolomé-Moreno

**Affiliations:** ^1^Department of Medicine, Psychiatry and Dermatology, Faculty of Medicine, University of Zaragoza, Zaragoza, Spain; ^2^Group B21-20R, Health Research Institute of Aragon (IISA), Zaragoza, Spain; ^3^Network for Research on Chronicity, Primary Care, and Health Promotion (RICAPPS) RD21/0016/0001, Zaragoza, Spain; ^4^Department of Psychology and Sociology, University of Zaragoza, Zaragoza, Spain; ^5^National School of Public Health (ENSAP), University of Medical Sciences of Havana, Havana, Cuba; ^6^Aragonese Healthcare Service (SALUD), Zaragoza, Spain; ^7^Department of Family and Community Care Teaching - Sector I, Aragonese Healthcare Service, Zaragoza, Spain

**Keywords:** meta-analysis, health literacy, primary health care, mental health, affective disorders, effectiveness

## Abstract

**Background:**

Affective disorders are a debilitating and very prevalent problem throughout the world. Often these are associated with the onset of comorbidities or a consequence of chronic diseases. Anxiety and depression are associated with poor social and personal relationships, compromised health. We aimed to synthesize evidence from studies measuring the impact of a health literacy (HL) intervention on the improvement of affective disorders.

**Methods:**

For this systematic review and meta-analysis, we searched PubMed/MEDLINE, Embase, Web of Science, Ibecs, Cuiden, Scielo, Science Direct and Dialnet for exclusively randomized controlled trial studies (RCTs) published between 1 Jan 2011, and 31 May 2022. The search terms employed were “health literacy,” “health knowledge,” “anxiety,” “anxiety disorder,” “depression,” “depressive disorder,” and “adult.” The risk of bias assessment was performed using the Cochrane Collaboration Revised Risk of Bias tool (RoB2). We conducted random-effects meta-analyses and explored heterogeneity using meta-regression and a stratified survey.

**Results:**

Of 2,863 citations found through the initial screening, 350 records were screened by the title and abstract for their themes and relevance. Finally, nine studies complied with the inclusion criteria for the meta-analysis. 66.66% of studies (*n* = 6) were rated as having a low risk of bias and 33.33% (*n* = 3) were judged to raise some concerns. The health literacy interventions were associated with −1.378 reduction in depression and anxiety questionnaires scores [95% CI (−1.850, −0.906)]. Low mood disorder scores are associated with better mental health and wellbeing.

**Conclusion:**

Our findings demonstrate that an HL intervention in relation to the symptoms associated with affective disorders improves the emotional state of patients in PHC, with a moderately positive effect in reducing depression and anxiety.

## 1. Introduction

Mental disorders are highly prevalence in our society (1). In 2019, the World Health Organization (WHO) reported that around 3.8% of people worldwide suffer from depressive moods and 4.1% suffer from anxiety disorders (2). So far, depressive moods and anxiety are included in the list of the main causes of morbidity in the world, and it is estimated that ~300 million people are affected (1). This figure has also increased considerably in light of the COVID-19 pandemic (3, 4).

In primary health care (PHC) settings, the prevalence of depression and anxiety are high. Patients with mental disorders or psychosocial conflicts are highly frequent users of health services. Nevertheless, depression and anxiety are very often underdiagnosed (5). A recent meta-analysis suggest that extremely low detection of depression was associated with a high value of increased severity of depression and suicidality (6). Therefore, the prevalence should be higher than that found, especially in the population at risk, i.e., women, widows, widowers, retirees, regular users of PHC services and those who have experienced stressful life events (7, 8).

Affective disorders are the result of complex interactions between social, psychological, and biological factors (9). Many of these mental health problems have a long duration and significant severity, which can alter the conciliation and daily life of the people who suffer from them, which generates great discomfort. The latest WHO reports on non-communicable diseases highlighted the importance of promoting better mental health and wellbeing (10, 11). Considering mental disorders are not deemed to be chronic health conditions by WHO, some suthors require to integrate mental health disorders into non-communicable diseases (12, 13).

Current evidence suggest the high probability of coexistence with chronic comorbid diseases, such as diabetes, hypertension, or cardiovascular diseases, may worsen affective disorders Furthermore, people with non-communicable diseases, such as diabetes mellitus, are at an increased risk of developing a mental disorder such as depression or anxiety (10, 14, 15). Mental disorders and cardiovascular diseases are the two main contributors to the global economic burden of non-communicable diseases and share a close relationship (16). In fact, a recent meta-analysis of 83 studies analyzed the pooled prevalence of depression and anxiety in adults with non-communicable diseases in Bangladesh, India, and Pakistan (17). As results, the prevalence of depression was 44% [95% confidence interval (95% CI) = (26, 62)], 44% for patients with chronic obstructive pulmonary disease [95% CI = (34, 45)], 39% for patients with diabetes [95% CI = (23, 56)], 38% for patients who had suffered a stroke [95% CI = (32, 45)], 38% for patients with hypertension, and 37% [95% CI = (30, 45)] for patients with cancer. In relation to anxiety, a prevalence of 29% is observed in patients with diabetes and 27% for patients with cancer (17).

Self-management of health is related to knowledge, self-care, self-coping abilities, and attitudes toward obstacles in life aside from purely biological conceptions (18). This would correlate with the concept of health literacy (HL), for which the first definition dates to 1998 (19): “Health literacy refers to the social and cognitive skills that determine the level of motivation and the ability of a person to access, understand and use information in a way that allows them to promote and maintain good health.” HL of communities and individuals has three basic levels: (1) health care and attention (accessing, understanding and interpreting medical-clinical information for informed decision-making); (2) disease prevention (correctly accessing, understanding and interpreting information about risk factors for health, and being able to make decisions regarding it); (3) health promotion (knowing health determinants, and being able to make lifestyle decisions accordingly) (20, 21). Community interventions in HL are particularly focused on modifying habits for a healthier life, closely related to the main health paradigms of Lalonde's determinants of health (22) and Dahlgren and Whitehead's Multilevel Model (23), with the Antonovsky's concept of salutogenesis (24) and Marmot's social determinants (25) or with the models of HL (20, 21, 26, 27).

Consequently, the level of HL has a direct impact on patients' ability to act and take on the medical-health information they receive, and on the real control that different individuals, families, or communities have over their own health (28). HL is lower in older people, social minorities, those with low socioeconomic status and those with limited access to health services (29–31). Moreover, greeting literacy have a high impact improving health and quality of life and healthy lifestyles (32). Currently, relevant evidence reported that health literacy leads to improved health knowledge, self-reported health status, shorter hospitalization, lower healthcare costs, and less frequent use of healthcare services (32–34).

The promotion of the participation of the population in coping with diseases and their self-care and self-management of health is a key element in the health of the general population and especially in the population with affective disorders. Disorders like anxiety or depression are associated with poor social and personal relationships, adverse life situations, compromised health and poor coping and problem-solving skills (7–9, 35).

Little evidence has been presented for the relationship between health literacy and affective disorders. Some studies reported that people with lower HL have more risk of mental illnesses and depressive symptoms (36–38). Moreover, low health literacy is associated with delays or failure to seek treatment for depression or anxiety. These delays have been linked to worse outcomes at the end of treatment (39, 40). However, little relevant evidence has been found of the effect of HL interventions on affective disorders.

Some authors suggest that there are cultural barriers, difficulties in accessing health services and a lack of a protocolised structure, which comprises a principal element of the interventions (41). These barriers also exist in accessing therapeutic alternatives. In fact, WHO has included “Implementing promotion and prevention strategies in the field of mental health” as a priority objective in its expanded mental health plan 2013–2030” (42).

The participation of the population in decision-making, in daily life, coping with diseases and the ability to self-manage health is promoted as a potentially key element in the future of the general population's health. Despite this, the role of HL interventions in the field of health, prevention and promotion of adopting resilient behavior despite mental health problems and responding appropriately to adverse situations is recognized. Our study aims to perform a systematic review and meta-analysis of the effect of HL interventions on more prevalent affective disorders (anxious and depressive symptomology) in adults in PHC.

## 2. Materials and methods

### 2.1. Search strategy

The present systematic review and meta-analysis study was conducted according to the Preferred Reporting Items for Systematic Reviews and Meta-Analyses (PRISMA) guidelines (43). A comprehensive search for HL interventions and their effect on depression and anxiety was undertaken using the PubMed/MEDLINE, Embase, Web of Science, Ibecs, Cuiden, Scielo, Science Direct and Dialnet databases. Moreover, a peer-review of relevant gray literature (e.g., economics working papers and academic theses) was performed. The search was conducted between 1 April and 1 June 2022. All articles published between 1 Jan 2011, and 31 May 2022 met the inclusion criteria. The search terms employed were “health literacy,” “health knowledge,” “anxiety,” “anxiety disorder,” “depression,” “depressive disorder” and “adult.” The reference lists of the included articles were also checked to find other appropriate articles. In Supplementary Table 1, we describe details of the search terms in full.

### 2.2. Conceptual framework

**Figure 1:**
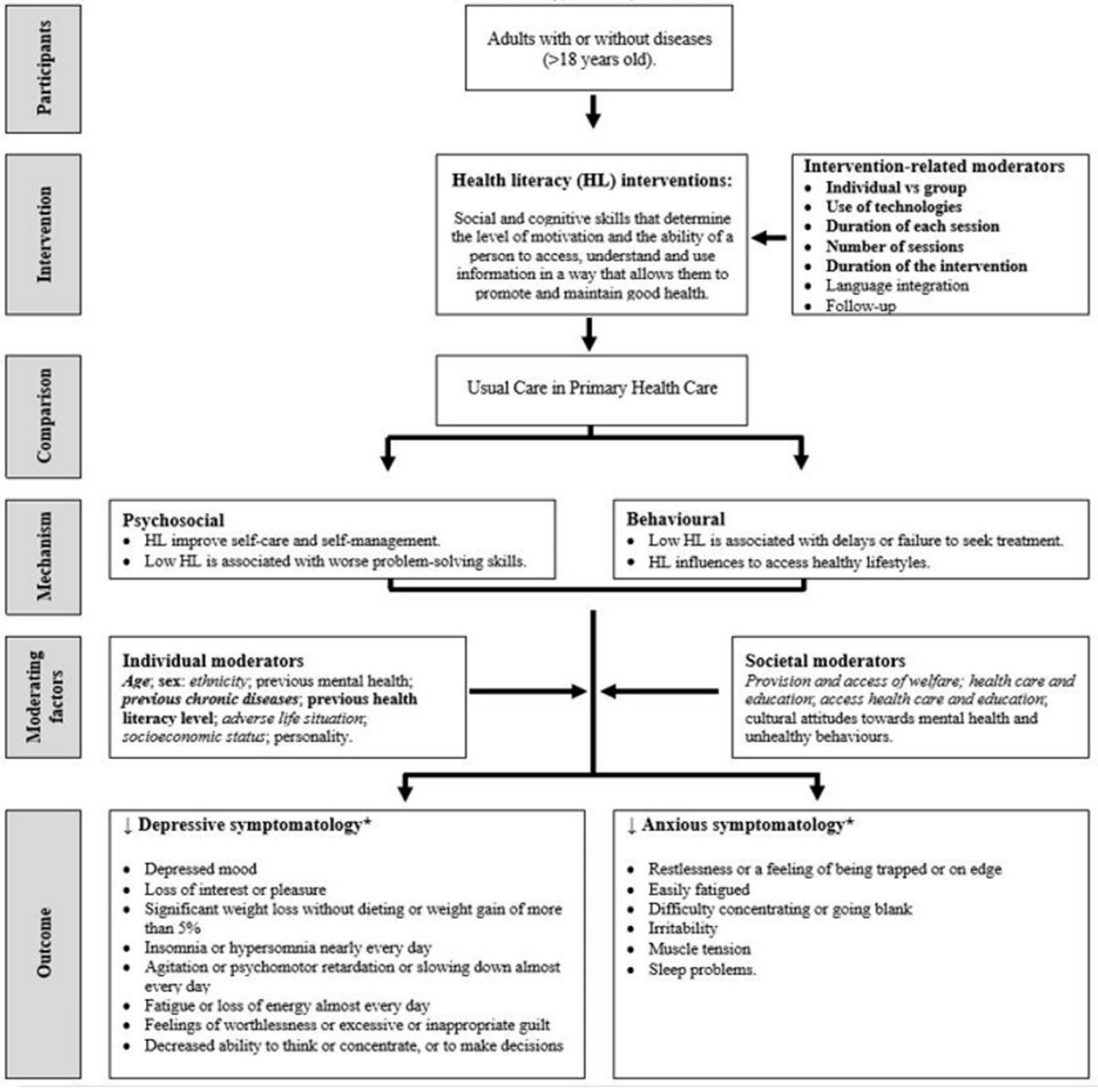
Conceptual model indicating theory of change for health literacy and mental health. ^*^Measured with validated with validated scales. Health literacy descripted following the model concept an definitions by Sorensen et al. (15); depressive and anxious symptomatology descripted following the definitions included in the DSM-V (38). Variables considered as important potential confounders in this study are indicated in italics and were selected *a priori* by the researchers based on variables that were viewed as key confounders in the literature. Variables considered important potential effect modifiers for exploration in this study are indicated in bold; these were selected based on assumptions regarding their likely importance and anticipated data availability.

In this systematic review, the research team developed a logic model according to the PICOS (Participants, Interventions, Comparison, Outcomes, Study design) principle (44). This model was developed to assist in the process of understanding and interpreting the effect of health literacy programs on better mental health and emotional wellbeing. In addition to identifying confounding factors and effect modifiers to explore in the subgroup analysis. The project team collaboratively developed the logic model, drawing on themes from the literature and the collective knowledge and experience of the team. The conceptual framework based on PICOS principle is presented in Figure 1.

### 2.3. Inclusion and exclusion criteria

The records retrieved were screened according to the inclusion and exclusion criteria. In this systematic review and meta-analysis, the inclusion criteria for the studies were as follows: (1) randomized controlled trials (RCTs) as the study design; (2) adults with or without diseases (>18 years old) as the population demographic; (3) health literacy interventions carried out in primary care whose main/secondary objective is to decrease mood disorder symptomatology as the intervention; (4) health literacy, depression and anxiety evaluated with validated scales as the methodology; and (5) scores of depression or anxiety scales baseline and post-intervention with their standard deviation or confidence interval as the outcomes. The study was also incorporated if the records presented the mean difference after the intervention, provided that it was accompanied by a standard deviation and/or confidence interval.

The exclusion criteria for the studies were as follows: (1) quasi experimental and observational studies; (2) studies with insufficient information or unpublished information; (3) studies with unspecified or incomplete methodology or the use of unvalidated scales; (4) studies in which the results were presented only in the form of graphs that did not allow the extraction of quantitative data; and (5) studies that were written up in neither English nor Spanish.

### 2.4. Data extraction and risk of bias assessment

A standardized Excel spreadsheet was prepared for data extraction. Two reviewers independently extracted and summarized the data according to the inclusion and exclusion criteria. A third reviewer resolved any disagreements through consensus or discussions. The information from each study included the first author, publication year, intervention title, country, sample size, patient demographics, type and characteristics of the intervention, scales outcomes (baseline mean, pre-post, and follow-up outcomes of depression and anxiety), length of intervention and follow-up. Finally, the data were rechecked by an independent author for accuracy. In the analyzed studies that presented different evaluations throughout the duration of the intervention, different types of intervention or different scales of evaluation of affective-emotional symptomatology were included in the meta-analysis as independent effects with the purpose of evaluating whether any of these specific characteristics could lead to a greater impact on mental and emotional wellbeing.

Certainty of evidence was assessed using the Grading of Recommendations Assessment, Development and Evaluation (GRADE) system (45). This combines information on five domains: risk of bias, imprecision, inconsistency (including statistical heterogeneity), indirectness (assessing how closely available data reflect the research question), and publication bias. A GRADE summary of findings is provided in the Supplementary Table 2. Also, the risk of bias assessment was performed using the Cochrane Collaboration Revised Risk of Bias tool (RoB2) (46). This tool is the recommended in Cochrane Reviews to assess the risk of bias in randomized trials included. RoB 2 is structured into a fixed set of domains of bias, focussing on different aspects of trial design, conduct, and reporting. Further detail on extracted items, decision rules, and RoB assessment, is available in the Supplementary Table 3.

### 2.5. Statistical analysis

For the meta-analysis, standardized mean difference (SMD) was employed. SMD measure of effect is used to report efficacy in terms of a continuous measurement (47). If the mean differences or standard deviation could not be directly extracted from the studies, they were estimated from the baseline and post-intervention outcomes, sample size, mean and 95% confidence intervals (95% CI), respectively.

We did a random-effect meta-analysis using models based on the DerSimonian and Laird method (Q-test) (48, 49). In addition we estimated the heterogeneity across studies using Higgins and Thompson method (I^2^ statistic) (50). Heterogeneity was considered statistically significant when I^2^ was more than 75% or the Q-test was < 0.1.

Random-effects meta-regressions were used to verify if the results were associated with any theoretical quantitative covariates (mean age, gender, sample size, and publication year), since these variables may explain the observed heterogeneity. To evaluate the impact of qualitative or dichotomous variables, the meta-analysis was stratified into different categories (chronic population, types of intervention, and improvement of HL following the intervention). The presence of publication bias was assessed using a funnel plot and Egger (51) and Begg's (52) statistical models. A *p*-value of < 0.05 was considered statistically significant. Openmetanalyst (53) and Stata v.15 software (54) was used for all of the statistical analyses.

## 3. Results

### 3.1. Literature search results

**Figure 2:**
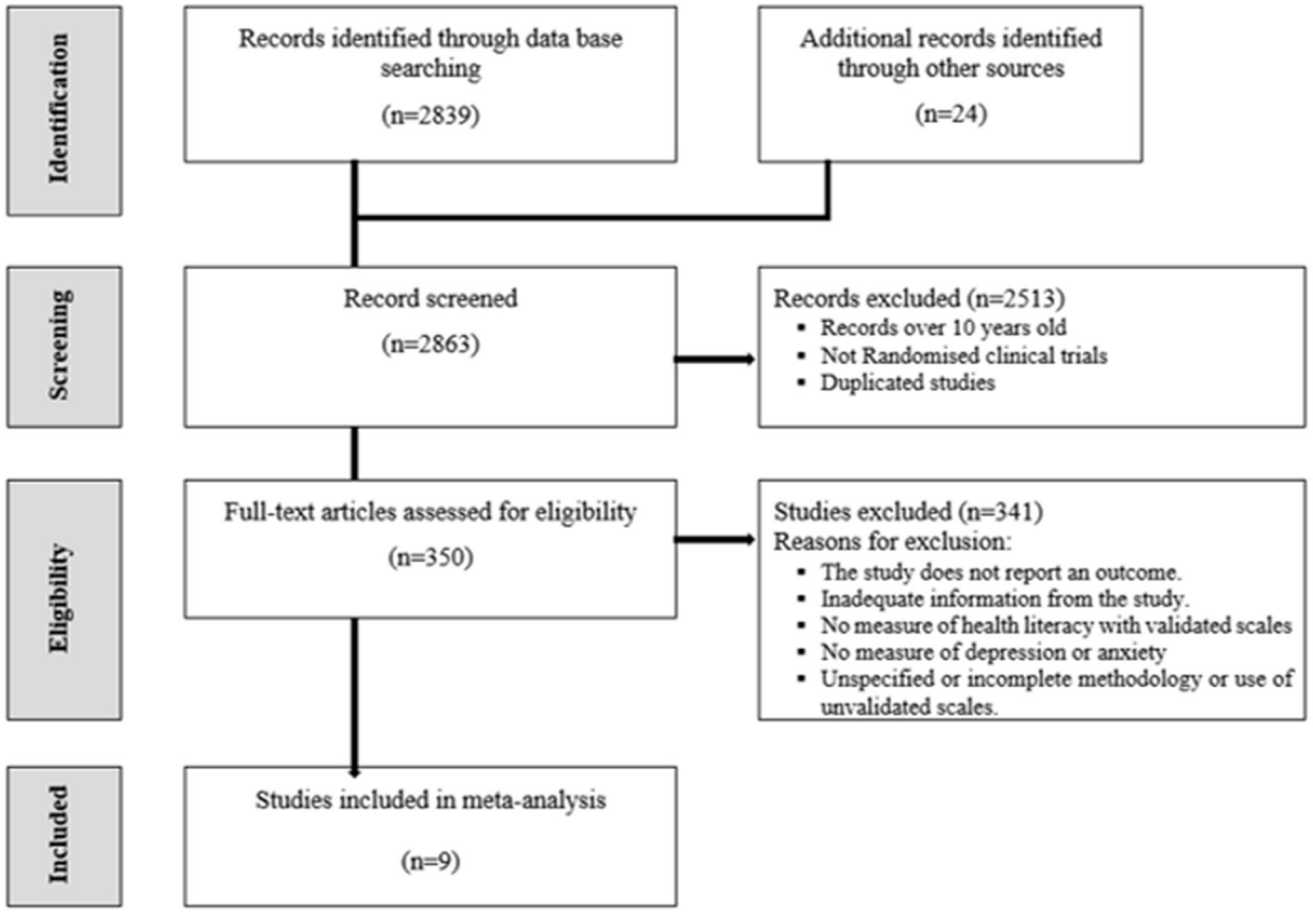
PRISMA flowchart process.

The full systematic search retrieved a total of 2,863 results. Following the removal of duplicate and non-RCT articles, 350 articles were screened by the title and abstract for relevance. Finally, nine studies (55–63) were incorporated into this meta-analysis to evaluate the association between HL interventions and depression and anxiety. The PRISMA Flowchart process is presented in Figure 2.

### 3.2. Study characteristics

All nine studies included (55–63) were RCTs with a total of 2,311 participants, of which 66.5% were women, and the mean age was 56.46. Four studies (55, 57, 58, 60) were conducted in Australia, two studies (59, 63) in the United States of America, one study (62) in Japan, one study (56) in Spain, and one (61) in the United Kingdom. All of the evaluated studies were published between 2011 and 2021. Table 1 shows a summary of the characteristics of the included studies.

**Table 1:** Summary of studies.

**Sociodemographic characteristics**	
Participants (*n*)	2,311
Mean age (years)	56.46
Female (%) (*n*)	66.55 (1,541)
Participants with chronic diseases (*n*)	4 studies (1,249)
Participants with depression at baseline (*n*)	2 studies (837)
**Evaluation**	
Only depression evaluation (*n*)	5 studies (1,064)
Only anxiety evaluation (*n*)	0 studies (0)
Anxiety and depression evaluation (*n*)	4 studies (1,247)
**Intervention characteristics**	
Interventions with more than two intervention groups	2 studies
Interventions applying literacy-adapted CBT	2 studies
Average participants of control group (min–max)	114 (30–302)
Average participants of intervention group (min–max)	142 (30–307)
Intervention with group sessions	3 studies
Intervention with digital monitoring	3 studies
Intervention with telephone call monitoring	2 studies
Average sessions (number) (min–max)	11.6 (2–30)
Average duration of each group session (minutes) (min–max)	68.33 (20–120)
Mean Intervention length (weeks) (min–max)	16.44 (2–52)
No follow-up	4 studies
Average length of follow-up (min–max) (months)	8.6 (1–12)

n, number of participants.

The descriptive characteristics of each study from our meta-analysis (author, year, country, sociodemographic, type of intervention, duration, characteristics of sessions, baseline scores) are presented in Table 2. The nine included studies had different scales of evaluation of the affective-emotional symptoms and health literacy. For depressive symptomatology, five studies used PHQ-9 scale (64), 2 studies GSD short version scale (65), one study used CESD scale (66), and the last one BDI-II scale (67). For anxiety symptomatology, only 4 studies evaluated anxiety and all of them with GAD-7 scale (68).

**Table 2:** Characteristics of included studies.

**References**	**Country**	**Sample size**	**Patient characteristics**			**Type of intervention**	**Session characteristics**	**Total sample baseline (mean ±SD)**	**Outcomes and follow-up**
			**Female % (** ***n*** **)**	**Age. mean** ±**SD**	**Patient**				
Bakker et al. (55)	Australia	*N* = 226 IG1 = 56 IG2 = 56 IG3 = 50 CG = 64	80.5% (182)	34.2 ± 12.1	Community people >18 years old	IG1:CBT-HL adapted app (activities, checker, a mood tracker, journal) IG2: self-monitoring mood-tracking app IG3: app that recommends CBT strategies CG: No intervention	Individual N° session = 30 Duration = 15–30 min	MHLQ-25 = 16.35 ± 2.26 PHQ-9 = 9.69 ± 6.07 GAD-7 = 7.08 ± 5.52	Baseline 4 weeks
Blancafort-Alias et al. (56)	Spain	N = 358 IG = 194 CG = 164	81.8% (293)	73.63 ± 6.9	Urban disadvantaged areas > 60 years	IG: Complex community program CG: No intervention	Group session (15 p per group) N° session = 12 Duration = 120 min	HLS-EU-16^*^; GDS-5 > 2: 59.69%	Baseline 12 weeks 12 months
Bohingamu et al. (57)	Australia	N = 171 IG = 86 CG = 85	47.9% (82)	70.41 ± 12.41	Diabetes and/or chronic obstructive pulmonary disease >18 years old	IG: Remote Patient Monitoring with an individual telehealth care plan CG: Usual care	Individual N° session = 2 Duration = 90 min	heiQ^*^; PHQ-9 = 6.595 ± 5.39 GAD-7 = 5.11 ± 5.41	Baseline 52 weeks
Heckel et al. (58)	Australia	*N* = 216 CG = 108 IG = 108	56.4% (122)	59.4 ± 12.2	Adults > 18 years who are cancer caregivers	IG: telephonic recommendations: psychological distress, health literacy, physical health, family support, financial burden, and practical difficulties CG: Usual care	Individual N° session = 3 Duration = 25 min	heiQ^*^; CES-D = 12.35 ± 0.91	Baseline 4 weeks 6 months
Johnson et al. (59)	United States of America	N = 228 IG = 95 CG1 = 71 CG2 = 62	57.7% (127)	59.33 ± 9.55	Patients with type 2 Diabetes mellitus and depression >18 years old	IG: Motivational and encouraging presential coaching. CG1: follow-up from their family physician. CG2: Usual care	Individual N° session = 8–12 Duration = 30–60 min	3QHL = 5.9 ± 2.6 PHQ-9 = 14.3 ± 3.6	Baseline 24 weeks 12 months
Kiropoulos et al. (60)	Australia	N = 202 IG = 110 CG = 92	50.5% (105)	65.40 ± 9.0	Greek-born or Italian-born first-generation immigrants >45 years old	IG: Online multilingual platform that content information about depression CG: No intervention	Individual online session N° session = 2 Duration = 90 min	D-Lit = 9.49 ± 3.75 BDI-II = 8.48 ± 7.83	Baseline 2 weeks 1 month
Salisbury et al. (61)	United Kingdom	N = 609 CG = 302 IG = 307	60.4% (417)	49.54 ± 12.8	Depression patients >18 years old	IG: A complex intervention incorporating use of technologies. CG: Usual care	Individual telephonic session N° session = 10 Duration = 20 min	eHEALS = 3.6 ± 0.8 PHQ-9 = 16.9 ± 4.6 GAD-7 = 12.95 ± 4.7	Baseline 16 weeks 8 months
Uemura et al. (62)	Japan	N = 60 CG = 30 IG = 30	66.6% (40)	74.00 ± 4.6	Lower health literacy patients > 65 years	IG: Active learning program focused on exercise, diet/ nutrition, cognitive activity, and health literacy CG: No intervention	Group session (5 p per group) N° session = 24 Duration = 90 min	HLS-14^*^; GDS-5 = 3.8 ± 2.9	Baseline 24 weeks
Van-Dyke et al. (63)	United States of America	*N* = 241 CG = 78 IGCBT = 83 IGEDU = 80	71.8% (173)	50.76 ± 8.7	Chronic Pain patients >18 years old	IGCBT: Literacy-adapted CBT IGEDU: pain Psychoeducation group CG: usual care	Group session (undefined) N° session = 10 Duration = 90 min	STOFHLA = 30.0 ± 7.5 PHQ-9 = 12.16 ± 6.5 GAD-7 = 9.09 ± 6.0	Baseline 10 weeks 6 months

N, total sample; CG, control group; IG, intervention group; SD, standard deviation; IG1, intervention group 1 (Moodkit); IG2, intervention group 1 (Moodprism); IG2, intervention group 3 (Moodmission); CG1, control group 1 (active control); CG2, control group 2 (usual care); HL, health literacy; CBT, Cognitive Behavioral treatment; IGCBT, Literacy-adapted group CBT; IGEDU, psychoeducation groups; MHLQ; mental health literacy questionnaire; PHQ, patient health questionnaire; GAD-7, general anxiety disorder; HLS-EU, European health literacy survey; GDS, Geriatric Depression Scale; heiQ, Health Education Impact Questionnaire; HLQ, Health Literacy Questionnaire; CES-D, Center for Epidemiological Studies Depression Scale; 3QHL, 3 Question of Health Literacy; D-Lit, Depression Literacy Questionnaire; BDI-II, Beck Depression Inventory; eHEALs, eHealth literacy scale; S-TOFHLA, Abbreviated version of the Test of Functional Health Literacy in Adults.

^*^Outcomes specific of Health literacy by domains.

For health literacy, 2 studies used heiQ scale (69), 1 study used Health Literacy Scale-14 (70), 1 study eHEALs scale (71), 1 study used HLS-EU-16 scale (72), 1 study used 3QHL questionnaire (73), and 1 study TOFHLA short version test (74). Moreover 2 studies evaluated health literacy with mental health literacy questionnaires [1 study used MHLQ-25 questionnaire (75, 76) and 1 study D-Lit questionnaire (77)]. All the scales used are itemized in the Supplementary Table 4.

### 3.3. Effect on mental health and mood disorders

For the meta-analysis of studies incorporated (55–63) we applied a random effects model to gauge the effect of HL interventions on mental health and mood disorders. According to our results, there was an observed moderately positive effect for reducing −1.378 points post scores [95% CI (−1.850, −0.906)] in depression and anxiety questionnaires. The decrease in the depression and anxiety scores implies the better mental health of the participants.

**Figure 3:**
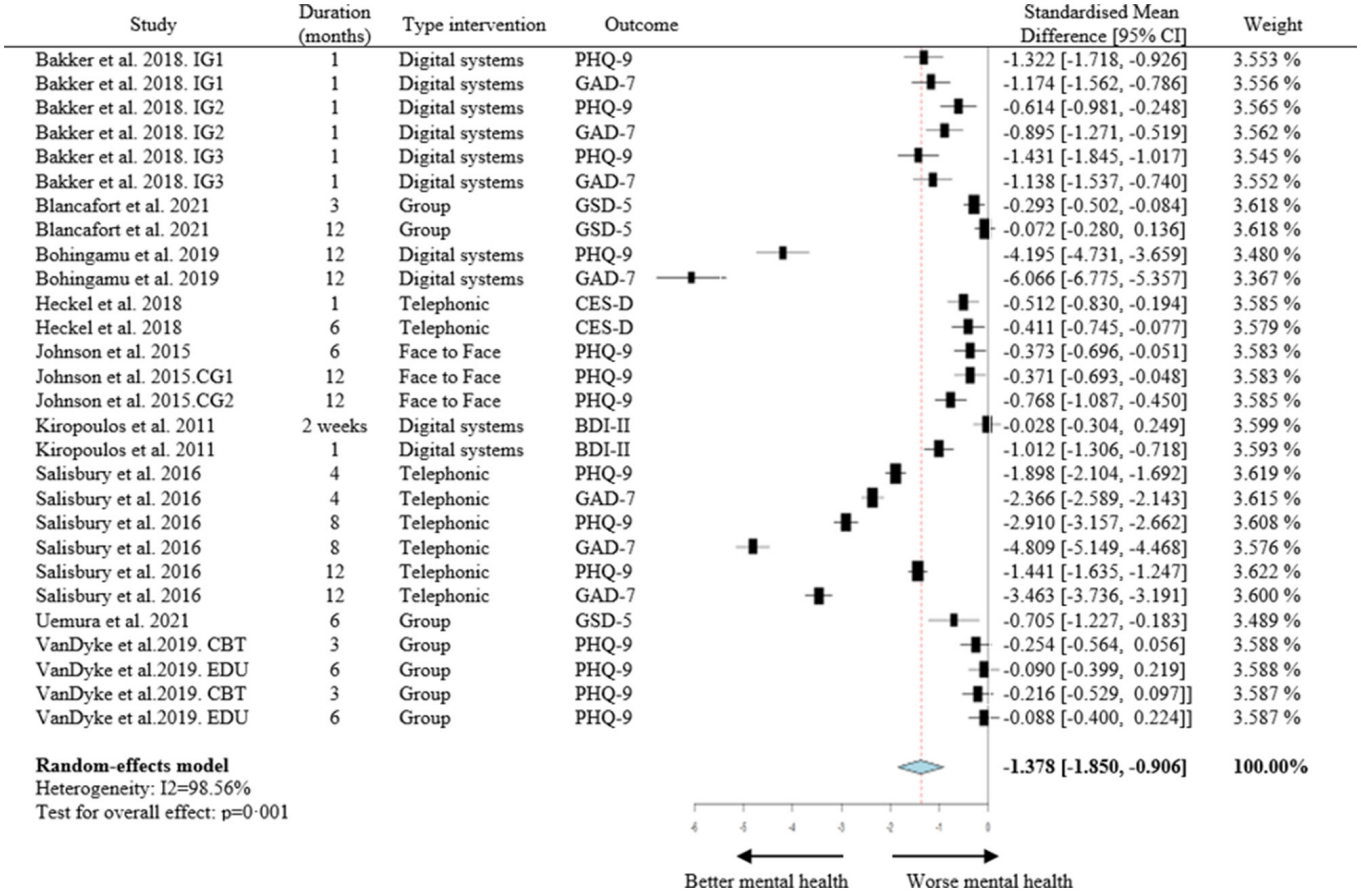
Forest plot for meta-analysis of studies reporting the effect on mood disorders. 95% CI: 95% confidence intervals; IG1, intervention group 1 (moodkit); IG2, intervention group 2 (moodprism); CG1, control group 1 (active control); CG2, control group 2 (usual care); CBT, cognitive behavioral treatment; EDU, psychoeducation groups; PHQ, Patient Health Questionnaire; GAD-7, general anxiety disorder; GDS, Geriatric Depression Scale; CES-D, Center for Epidemiological Studies Depression Scale; BDI-II, Beck Depression Inventory.

If we differentiate the effect of the type of affective-emotional symptomology, the anxious symptomatology decreased by −2.829 points [95% CI (−3.981, −1.676)], vs. depressive symptomatology that decreased by −0.897 points [95% CI (−1.295, −0.499)]. However, it is necessary to point out that none of the studies included have evaluated the effect on anxiety as the only mental health variable. The effect sizes for each RCT researched in our meta-analysis are displayed in **Figure 3**.

In Figure 3, the effect obtained in the RCTs that carried out different evaluations during the study has been included independently, as in Salisbury et al. (61). Moreover, the independent effect was also considered if the study presented different types of intervention within it, such as the studies of Bakker et al. (55) or Van-Dyke et al. (63). On the other hand, for the studies that evaluated both depression and anxiety symptoms, as suggest in the study by Bohingamu et al. (57), the effect of each score is incorporated independently in this analysis. Heterogeneity among the studies that we had included was I^2^ 98.56% and Q was a *p*-value < 0.001, which is highly significant. This heterogeneity indicated a high variability in the characteristics of the different interventions analyzed. To obtain an exact prediction of the patients who will benefit from better mental health with HL interventions, a stratified analysis is performed through subgroups and meta-regression.

### 3.4. Effect according to the type of intervention

The outcomes of the type of intervention reported that individual interventions decreased the mood disorder symptomology more than group interventions [−1.757; 95% CI (−2.309, −1.206)] vs. [−0.197; 95% CI (−0.311, −0.082)], respectively, as shown in Table 3. When comparing the type of individual therapy, telephonic interventions obtained better results in anxious and depressive symptomology vs. face-to-face interventions or interventions using digital platforms.

**Table 3:** Effect of the type of intervention on affective symptomatology.

**Subgroups**	**Number of studies**	**Difference of mean (95% CI)**	**I^2^ statistic**	**Q-test**
**Individual/group**				
Individual	6	−1.757 (−2.309, −1.206)	98.52%	0.000
Group	3	−0.197 (−0.311, −0.082)	13.27%	0.329
**Individual intervention**				
Face to face	1	−0.505 (−0.765, −0.245)	48.12%	0.140
Digital systems	3	−1.756 (−2.551, −0.962)	97.66%	0.000
Telephonic	2	−2.226 (−3.074, −1.378)	98.88%	0.000

95% CI, 95% confidence interval; I^2^ statistic, I^2^ test Higgins and Thompson; Q-test: Q test Der Simonian and Laird.

In relation to this analysis by subgroup, individual interventions maintain high scores for Heterogeneity I^2^ and Q when compared to group interventions [(98.52%; 0.000) vs. (13.27%; 0.329), respectively]. Furthermore, the heterogeneity of the different types of individual interventions was also significant, except in the case of face-to-face interventions. These results indicate that there is variability in different individual interventions, while group interventions do maintain uniformity in their intra-study characteristics.

### 3.5. Effect according to chronic pathologies

Our meta-analysis contains four RCTs (57, 59, 61, 63) focused on the effect of HL in patients with chronic pathologies. As shown in Table 4, we reported a greater decrease of mood disorder symptoms in the demographic with chronic pathologies [−1.938; 95% CI (−2.679, −1.196)] compared to the community population [−0.722; 95% CI (−0.948, −0.459)]. Digital systems interventions obtained a better effect for reducing depression and anxiety scores in patients with noncommunicable diseases [−5.115; 95% CI (−6.949, −3.282)] vs. face-to-face [−0.505; 95% CI (−0.765, −0.245)] or telephonic interventions [−2.808; 95% CI (−3.658, −1.958)]. In relation to this analysis by subgroup, both groups maintained a high heterogeneity I^2^ and Q [(99.24%; 0.000) and (84.71%; 0.000), respectively]. These results indicate that there is high intra-study variability in both conditions.

**Table 4:** Effect of the chronic condition of the participants on affective symptomatology.

**Chronic patients**	**Number of studies**	**Difference of mean (95% CI)**	***I*^2^ statistic**	***Q*-test**
Yes	4	−1.938 (−2.679, −1.196)	98.99%	0.000
No	5	−0.722 (−0.984, −0.459)	88.45%	0.000

95% CI, 95% confidence interval; I^2^ statistic, I^2^ test Higgins and Thompson; Q-test, Q test Der Simonian and Laird.

### 3.6. Effect of the significant improvement in health literacy

Five (56, 57, 60–62) of the included studies obtained a statistically significant increase in HL level as a result of their intervention. Two studies reported an improvement of some domains of HL as Skill and Technique Acquisition (heiQ scale), while another study reported an increase in the Communicative Health Literacy (Health Literacy Scale-14). Other studies only report final scores and are undivided by domains (eHEALs scale, D-Lit questionnaire). Due to the use of different health literacy scales with different scoring systems, it was decided not to incorporate the baseline or post-intervention mean of these scales as a continuous variable. Instead, the significant increase in LH post-intervention was included as a dichotomous variable, as shown in Table 5. The effect of each intervention on the HL values of the included studies can be seen in Supplementary Table 5.

**Table 5:** Effect of the significant improvement in health literacy on affective symptomatology.

**Improvement of HL significant**	**Number of studies**	**Difference of mean (95% CI)**	***I*^2^ statistic**	***Q*-test**
Yes	5	−2.233 (−3.041, −1.425)	99.16%	0.000
No	4	−0.629 (−0.844, −0.413)	83.18%	0.000

HL, health literacy; 95% CI, 95% confidence interval; I^2^ statistic, I^2^ test Higgins and Thompson; Q-test, Q test Der Simonian and Laird.

To report whether this significant increase can influence affective symptomatology, an analysis was performed per subgroup. Studies with a significant improvement of HL obtained better outcomes in mood disorder symptoms [−2.233; 95% CI (−3.041, −1.425)]. The results are recorded in Table 5. In relation to this analysis by subgroup, both groups show a high heterogeneity.

### 3.7. Meta-regression

As Figure 3 shows, the heterogeneity assessed by the I^2^ statistic and Q test among the included studies was 98.56% and *p*-value < 0.001, respectively. Due to the high variability in the characteristics, it was verified whether some theoretical covariates (mean age, gender, sample size and intervention duration) served as cofounders that could affect the results. The results of meta-regression showed that affective symptomatology scores were significantly higher when the sample had more women than men (*p*-value < 0.05). This condition is very influential in those studies whose target population was chronic patients (*p*-value < 0.001). Among the results found, it is important to highlight the influence that the increase throughout the total duration of the intervention has on the better decrease in affective symptoms (*p*-value < 0.001), as shown in Table 6.

**Table 6:** Meta-regression.

	**Effect on mood disorders**		**Effect on individual interventions**		**Effect on Chronic Patients**		**Effect on interventions with HL significative**	
	**Coefficient (95% CI)**	**P-value**	**Coefficient (95% CI)**	**P-value**	**Coefficient (95% CI)**	**P-value**	**Coefficient (95% CI)**	**P-value**
Age	−0.026 (−0.044, 0.096)	0.470	−0.038 (−0.088, −0.012)	0.132	−0.090 (−0.198,0.018)	0.102	−0.130 (−0.402, 0.142)	0.349
Sample size	−0.003 (−0.008, 0.002)	0.184	−0.002 (−0.005, 0.002)	0.317	−0.003 (−0.007, 0.002)	0.294	−0.012 (−0.029, 0.004)	0.142
%Female	**0.051 (0.009, 0.093)**	**0.016** ^*^	**−0.208 (−0.396**, **−0.019)**	**0.031** ^*^	**0.152 (0.066, 0.238)**	**0.000** ^*^	**0.155 (0.022, 0.287)**	**0.002** ^*^
Session min	0.006 (−0.024, 0.037)	0.682	−0.008 (−0.032, 0.017)	0.541	0.014 (−0.015, 0.044)	0.337	0.020 (−0.002, 0.042)	0.069
No sessions	−0.005 (−0.106, 0.095)	0.922	0.041 (−0.017, 0.099)	0.165	**0.341 (0.130, 0.552)**	**0.002** ^*^	0.065 (−0.025, 0.254)	0.108
Duration intervention	**−0.081 (−0.116**, **−0.047)**	**0.000** ^*^	−0.061 (−0.093, −0.029)	**0.000** ^*^	**−0.072 (−0.123,−0.021)**	**0.000** ^*^	−0.067 (−0.113,−0.022)	**0.000** ^*^

%, Percentage; No, number; min, minutes; HL, health literacy; 95% CI, confidence interval 95%; ^*^, statistically significant.

Bold values are statistically significant.

This evaluated characteristic influences all of the intervention subgroups and characteristics of our sample population, being able to highlight the notable influence that this variable has on the study being carried out. Additionally, in individual interventions, depression and anxiety symptomatology scores decreased significantly with the improvement of time for each session (*p*-value < 0.05). As Table 6 shows, the variables that may be influencing this heterogeneity are the proportion of women in the sample, the duration of the intervention and the number of the sessions carried out.

### 3.8. Certainty, and risk of bias assessment and publication bias of the included studies

The certainty of evidence assessment was performed using the Grading of Recommendations Assessment, Development and Evaluation system. We report with high certainty that individual interventions have beneficial effects on mental health and wellbeing (e.g., an individual telephonic intervention is associated with a high decrease of depression and anxiety score). However, there were three studies (58, 60, 63) with low certainty due to serious inconsistency (heterogeneity; I^2^ >75% in meta-analysis) and to suspected negative influence of Residual Confounding. Our estimates of effect size for key outcomes were subject to high, moderate, or low certainty. The certainty assessment of each study can be seen in Supplementary Table 2. Moreover, the risk of bias assessment was performed using the Cochrane Collaboration Revised Risk of Bias tool. among the nine randomized controlled trials six were judged to have an overall low risk of bias, three (55, 59, 60) were judged to raise some concerns for bias, and none of them were judged as having a high risk of bias. The risk of bias assessment of each study can be seen in Supplementary Table 3.

**Figure 4:**
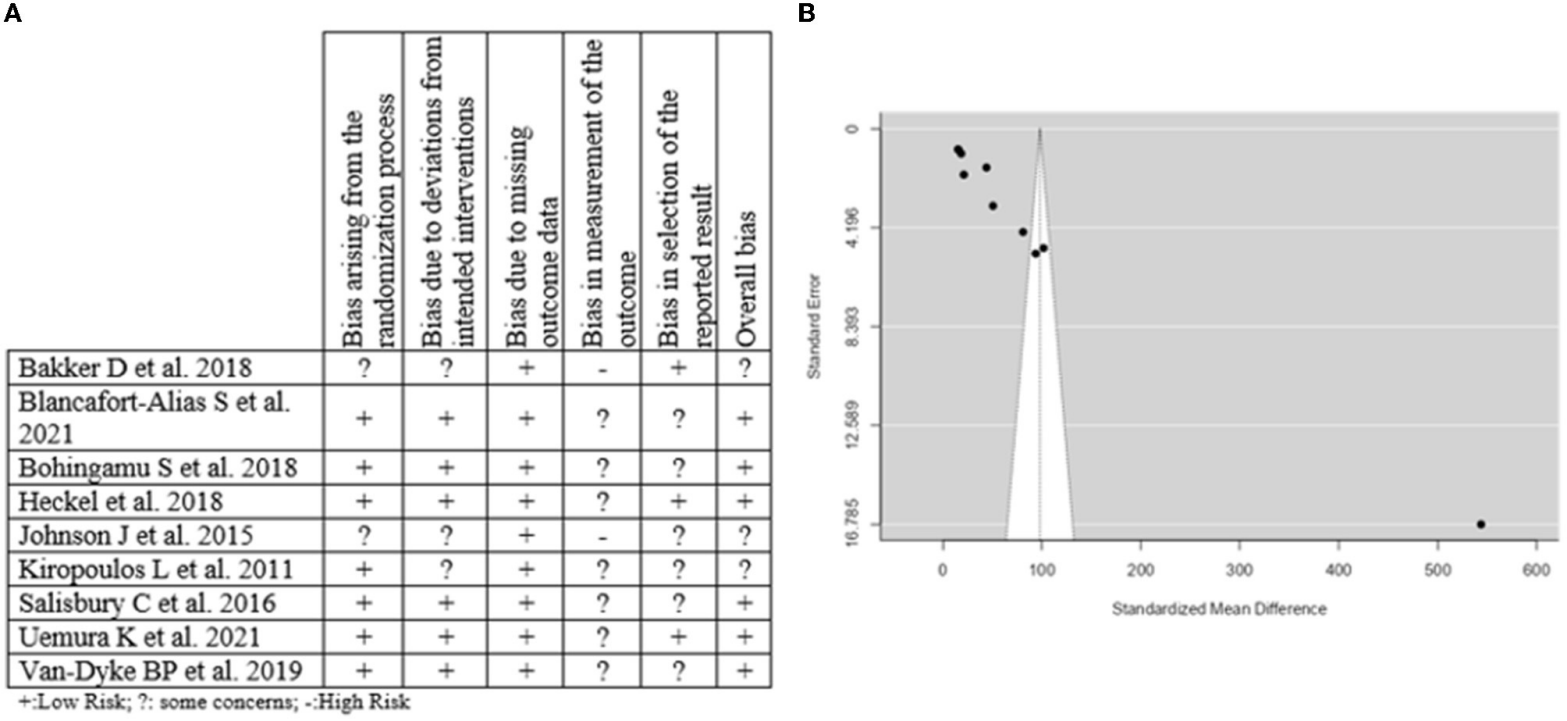
Risk of bias assessment and publication bias of the included studies. **(A)** Risk of bias assessment. **(B)** Funnel plot.

A question of assessing risk was the domain related to the outcome measure. This finding could be explained by the absence of or the presence of only simple blinding of the evaluated studies, which generates bias in the results obtained. Several of the studies also report this in their limitations. The risk of bias assessment is presented in Figure 4A. Alternatively, publication bias was significant in the analyzed studies (*p* < 0.001 in the Begg's test, *p* < 0.001 in the Egger's test) and with the funnel plot shown in Figure 4B. Given what was obtained in the meta-regression, it can be surmised that there is a relationship between the existence of publication bias and one of the most essential characteristics of the interventions, which is the duration.

## 4. Discussion

The aim of this systematic review and meta-analysis is to evaluate the effectiveness of HL interventions in the improvement of anxiety and/or depressive symptomatology in adults in PHC.

So far, not enough evidence has been presented in studies with the same objective as ours, so our review and meta-analysis allows for a new evidence-based approach regarding this issue. Our results support the effect of HL interventions in improving mood by reducing symptoms of depression and anxiety in PHC patients. This is because HL interventions are more effective in populations with previous chronic pathologies and in individualized and lengthier interventions.

### 4.1. Effect according to the sociodemographic characteristics of target population

In our meta-analysis, age was not a significantly influential variable on the effect of individual interventions, with affective symptomatology decreasing less in older participants than in younger participants. The mean age in our study was 56 years old. However, the mean age in the studies on individual interventions has greater variability than in the studies on group intervention (34.2–70.41 vs. 50.76–74, respectively). Few studies referred to a systematic review of interventions in HL in older people since they worked with less restrictive criteria than ours regarding the role of health information in promoting the health and wellbeing of older adults (78). Watkins et al. (79) emphasized the need for researchers to develop and assess high quality interventions in e-health literacy interventions targeting the older population, among whom there is a general lack of knowledge. Following our analysis, the authors of this study reinforce the necessity of improving the availability and accessibility of information for self-management and individuals' HL skills. Recently, Uemura et al. (62) examined the effects of an active learning program in HL, lifestyle behaviors, physical function, and mental health among older community-dwelling adults with low HL. The intervention group demonstrated a significant improvement in communicative HL and depressive symptoms among other variables.

Overall, the published studies that coincide with our outcomes show a greater responsiveness and effect in the young and adolescent population, perhaps in relation to the introduction of e-health tools (80, 81). Even one of the studies, by Bakker et al., attained exceptional results in improving affective disorders in patients with anxiety and baseline depression, with a mean age of 34 years old (55, 82, 83).

Concerning sex and gender, our study found, as in other published studies (84), that there is a higher baseline prevalence of affective disorders in women than in men. Of the 1,541 women included in our study, 35.30% had been diagnosed with depression at the beginning of their intervention. In our study, the studies in which the percentage of women was greater than men in the sample showed that the effect of the reduction of symptoms was less, especially in the studies in which the population comprised patients with chronic conditions (*p* < 0.05 and *p* < 0.001, respectively). This finding could be explained by the fact that, indirectly, the effect of the intervention in the trials with a higher percentage of women, and therefore of baseline affective disorders, was lower. Through the review of the current evidence, we have not found RCTs that explore the perspective of sex or gender in relation to the effectiveness of interventions.

### 4.2. Effect according to the intervention

In relation to the content included in the interventions and their focus on mental health and affective symptomatology, the results obtained did not suggest that interventions with a focus on mental health obtained better results in affective-emotional symptomatology in the main analysis.

Four of the included studies treat depressive symptomatology as a primary outcome, and the 5 remaining studies treat it as a secondary outcome. Only 2 studies had mental health as the focus of the intervention (55, 60), 6 studies treated mental health as a topic concerning the set of sessions of the intervention (56, 58, 59, 61–63), and 1 study carried out an individualized intervention focusing on the patient (57). Thus, the topics of the intervention in health literacy were not pre-established and depended on each patient.

In the meta-analysis, it was observed that individual interventions were more effective than group interventions; however, greater uniformity has been found in group interventions compared to the heterogeneity of individual ones. One of the common problems shared with other group interventions is a lack of adherence to heterogeneous factors, especially in disadvantaged socioeconomic contexts and among older people. An example of this situation can be found in the study on community samples by Blancafort-Alias et al. (56), who carried out an intervention in SA with a strong social component and measurements at 3 and 12 months in disadvantaged populations in PHC. This study showed that the intervention was effective in improving the mental health of older adults in disadvantaged urban areas, and the results of this study provide evidence to policymakers on how to promote health, with an emphasis on salutogenesis by promoting self-management and health literacy (85).

Currently evidence suggests that HL interventions have started using new technologies as instruments for improvement. This can be observed in the studies collected in our review: 3 out of 9 studies used digital technologies exclusively (55, 57, 60). In patients with underlying chronic diseases, digital interventions obtained better results than face-to-face interventions. In addition to the general population or patients with depression (61), the implementation of means for improving HL through telematic services has been used, for example, in patients with breast cancer (86). This study also analyzed changes in the anxiety and depression scale score after chemotherapy. Other outcomes included HL (measured using the 14-item HL scale). However, no significant improvement was observed in patients with anxiety and depression or in HL using smartphone applications or control groups at the end of treatment (86).

Salisbury et al. (61) evaluated the effectiveness of an integrated telehealth service in 43 British health centers for 608 patients with depression. When compared to usual care alone, intervention participants reported improvements in their anxiety as well as better access to and satisfaction with the support they received, and improvements in self-management and HL. Nevertheless, they found only a slight improvement in the mean global score of the PHQ-9 for depression. The authors have suggested that a worse response may occur in the context of chronic depressive patients treated in PHC, which may explain any poor responses to non-pharmacological treatment (61, 87).

Bakker et al. (55) designed an RCT that compared the efficacy of three publicly available apps for mental health in a young community sample, and there were significant and homogeneous results in all aspects. All users of the mental health applications experienced an increase in mental wellbeing and a decrease in depression and anxiety when compared to the control group. Interestingly, the greater involvement of the users in the use of e-health literacy tools had a greater impact on the reduction of depression, anxiety, and better levels of emotional wellbeing than those who did not rate their engagement at all. Emotional self-awareness only influenced the effect of improving mental health in participants who were clinically depressed or anxious at the time of the baseline assessment (55). In this regard, it has been theorized that mental health apps may exert effects on mental health and wellbeing through mental health literacy (MHL) and self-efficacy through coping mechanisms (88, 89). MHL can be improved by providing access to information on mental health and psychoeducation as well as by giving people tools to gain self-confidence in their own ability to cope with distress and adversity (90, 91).

The integration of language in the intervention is also a relevant factor in achieving the objectives of any intervention. The approach to HL in certain population groups has been investigated in various studies (60, 92, 93). Also fulfilling the inclusion criteria, the RCT of Kiropoulus et al. (60) was evaluated to investigate the effects of the multicultural information provided by e-health on depression in Greek and Italian immigrants in Australia. The intervention group showed higher depression literacy scores post-assessment (*p* < 0.001) than those in the control group. The results suggest that the internet may be a feasible and effective means to increase knowledge of depression and decrease personal stigma. In contrast, the lack of change in perceived stigma in this trial is consistent with results in other trials examining online depression stigma interventions in English-speaking groups. The authors explain that an important limitation of the study was that the post-testing and follow-up testing occurred shortly after the completion of the intervention (60).

Another important factor in the intervention is the team that applies it in each study. 3 studies detailed that the intervention was applied by a team made up of general practitioners and nurses. 1 study detailed that the intervention was requested by a nursing team, while another was performed using physical therapy. The remaining four studies did not specify the type of professional that applied the intervention; it was only implied that the research team of the study performed this intervention. Although the results obtained suggest that the interventions carried out by the nursing and medical teams in primary care effected an improvement in mental health, due to the lack of sufficient information regarding the different studies, the overall study does not allow for a good analysis of the influence of the interdisciplinary team in obtaining better results in mental health.

### 4.3. Effect according to chronic pathologies

Our meta-analysis demonstrates that interventions in HL are more effective in improving affective disorders in patients with certain chronic pathologies than in the general population, although improvements in mental health are evident in both groups. There are research groups that have focused on seeking evidence from interventions in people with non-communicable diseases by even creating specific measurement scales (94–96). Unfortunately, many publications were excluded in this meta-analysis because they did not provide quantitative data following the intervention. When specific interventions are scheduled for a given morbidity, they are usually aimed at improving the dimensions of HL related to knowledge and self-care of a specific pathology. This type of intervention can be carried out individually or in a group.

This was the case in four of the studies included in our meta-analysis that specifically addressed chronic pathologies. Bohingamu et al. (57) assessed the impact of home-based telehealth monitoring on health outcomes, quality of life, and costs for patients with diabetes and/or chronic obstructive pulmonary disease. The intervention group (86 vs. 95 for the control group) showed a significant improvement in anxiety, depression, and HL at 12 months, which comprises the improvement in the mental health of patients with chronic pathologies (57).

A study included in our meta-analysis, which looked at highly prevalent chronic diseases such as diabetes, performed this RCT in 14 PHC clinics. The study included 214 participants with poorly controlled diabetes and/or coronary heart disease alongside coexisting depression. In addition to their usual care, an intervention involving nurses who provided guideline-based, patient-centered management of depression and chronic disease significantly improved the management of patients' medical condition(s) and depression. While these authors only researched these specific methods, collaborative care was also shown to improve depressive symptoms (59, 97).

The same group of researchers made a hypothesis based on a substudy on the association of inadequate HL with health outcomes in patients with type two diabetes and depression (98). The exposure of interest was inadequate HL, measured by a brief scale comprised of three questions (73). Curiously, participants in both groups saw significant and important improvements in their depressive symptoms from baseline to 12 months, except in PHQ-9 scores between the groups. Using the random-effects model, the adjusted difference in the average change of PHQ-9 scores was neither statistically significant (*p* < 0.482) nor clinically relevant, and there were no statistically significant differences in any outcomes between the HL groups (98).

Heckel et al. (58) conducted a telephonic intervention in which the depression and HL level of caregivers of cancer patients were measured as secondary variables. For caregivers at risk of depression, the intervention had a significant effect on their confidence in having sufficient information to manage their health.

Through studies on a chronic pain population, Van-Dyke et al. found that educationally or cognitively disadvantaged patients benefit most from the more structured approach of literacy-adapted cognitive behavioral therapy rather than psychoeducation, whereas less disadvantaged patients benefit from treatment through the outcome variable, which is improvement in chronic pain (63, 99). In relation to our meta-analysis, it was observed that the studies that demonstrated a significant increase in HL level post-intervention were related to better scores in affective symptomatology. However, when specifically analyzing the effect on depression as a secondary outcome when compared to the three interventions in patients with chronic pain, it was found that changes in depression did not differ between the cognitive therapy adapted to HL and the educational or usual care group; in essence, there were no differences between the three groups (63, 100).

In relation to the evidence of other chronic pathologies, Nesbit et al. examined the effect of an intensive health education and counseling intervention to improve self-care in 614 rural patients with heart failure (HF). The results were that the severity of HF, worse HF knowledge, poorer perceived control, and symptoms of depression or anxiety were associated with a worse perception of quality of life in patients with HF in rural areas (101). Within the same project, intervention was evaluated as a secondary variable among randomized cardiac patients with and without depression. No intervention effects were observed in patients with depressive symptoms but significant improvements in self-care of their heart disease were found in patients without baseline depression (102). These results have been supported by a recent systematic review by the same group of researchers and suggest that depression is the only factor consistently reported to be associated with poor self-care in patients with HF (103, 104). In both studies, the interventions in HL were not specifically designed to improve the symptoms of mental discomfort that patients might have (101–103).

The same authors evaluated the level of knowledge regarding self-care in patients with chronic conditions and psychological distress. In this study, patients with better knowledge and self-care tended to have worse HF grades, higher depression and anxiety, and lower levels of perceived control. This corresponds with the claim in the previous study that the intervention in HL has no effect on patients with depression but does on non-depressed patients (105). One explanation may be the fact that individuals with depression often experience short attention spans, lack of motivation, loss of energy, and even psychomotor retardation, all of which contribute to difficulties in learning about their chronic illness and possible self-care strategies. The intervention effect can be enhanced by reinforcing their learning and encouraging motivation when their depressive symptoms are in remission. Social support is another potential key factor in improving self-care in HF patients with comorbid depression, and its lack of social support contributes to both depression and poor self-care (102).

The study in question also examined the possible association between degree of literacy and cognitive impairment, specifically in a sample of 226 people over 65 years of age. 37% of the participants showed limited health literacy, directly related to a greater deterioration of the executive functions at the 12 month evaluation, demonstrating that low HL is related to the further deterioration of cognitive functions (106).

Closely related to chronic pain, it was found that this RCT, in which a planned HL intervention program was analyzed in comparison with an intervention directive between oncological and non-oncological patients, whose secondary outcomes were depression and/or anxiety and HL, among others. Ease of use of the documentation and satisfaction were high, and depression/anxiety was low, with no differences based on the study arm or whether the patient had cancer or not (107). In another geographical context and with the same objective, the prevalence of symptoms of depression and anxiety and mental health literacy (MHL) in outpatients with or without cancer was evaluated. MHL was comparable between oncology patients and controls and was positively associated with their level of education. Thus, the data suggests a strong association between level of education and MHL: those with higher education were more likely to possess psychological mindedness and better help-seeking knowledge (108).

### 4.4. Heterogeneity, risk of bias, and risk of publication

In our meta-analysis, there was a significantly high degree of global heterogeneity assessed by the I^2^ statistic (50) and Q test (48) (98.56% and *p*-value = 0.001 respectively). Thus, there was a lot of variability in the samples and in the interventions, especially as far as individual interventions are concerned. The effectiveness of the RCTs examined was highly variable, even in the same RCT throughout the follow-up, as in Blancafort-Alias et al. Other studies have shown a very narrow confidence interval in their results, which suggests high homogeneity.

Given the high degree of heterogeneity, a meta-regression analysis was necessary. As observed in the results of the meta-analysis, the heterogeneity in the group interventions was less than in the individual ones. This fact could be associated with the smaller disparity between the durations of the interventions: the duration of the group interventions were 3–6 months, while the individual interventions varied between 2 weeks and 12 months. This allowed us to observe that interventions based on HL varied according to the problem requiring treatment and the characteristics of the population to which it was directed. Although there is no consensus on how to conduct these interventions to obtain the greatest benefit for the population, as is apparent in the results of the analysis, longer lasting interventions obtain better results (109, 110).

As reported by the analysis, the proportion of women influenced the heterogeneity obtained not due to the proportion of female participants in the studies (66.55%) but rather the presence of previous depression in the total population included (23.5% of women vs. 12.6% of men) (84).

The results of the risk of bias assessment were due to the lack of blinding in these three studies (55, 59, 60). Some research groups reported in their results that, despite having a single blind, interactions had been detected between the participants in the arms of the trial, which may have distorted the results. However, in relation to the publication bias and what was obtained in the meta-analysis results, there is a relationship between this publication bias and one of the essential characteristics of the interventions. This variability between the basic characteristics of the interventions, such as the duration of the intervention and the evaluation tools, are associated with this high publication bias (110, 111).

### 4.4. Limitations

Some limitations of the meta-analysis should be mentioned. The first of these is the somewhat strict selection criteria. Ultimately, this meant that only nine RCTs were included, although the total sample (2,311 patients) was considered sufficient. Over the last decade, there have not been any more RCT studies published that investigate the effect of an intervention in HL concerning emotional wellbeing and meet the inclusion criteria. However, recent research has been considered more reliable for this study as the temporality of RCTs in this type of intervention was relevant compared to those that were published over 10 years ago and thus discarded in this study. Another limitation was the difficulty of evaluating some RCTs due to the lack of quantitative data, which is an essential requirement for meta-analysis. However, some relevant studies that did not meet the inclusion criteria were incorporated into the discussion. In relation to the publication dates of the studies, 2 studies were published in 2021 and may have been affected by the COVID-19 pandemic. However, a review of their methodology and records showed that they were carried out in 2017 and 2018; thus, the results were not influenced by the COVID-19 pandemic (56, 62). Furthermore, the fact that anxiety and depression have increased during the COVID-19 pandemic must be taken into account.

In the meta-analysis, it was found that some of the studies included emotional distress variables (anxiety and depression) as secondary variables of their intervention and not as the main outcome, which may have conditioned the analysis and results. Additionally, no RCTs have investigated the effect of HL on isolated symptoms of anxiety, which is one of the most frequent affective disorders in PHC. The long-term impact of HL intervention is unknown, and our meta-analysis has not filled this gap in the literature (78).

In the meta-analysis, it was not possible to quantitatively compare the HL baseline or post-scores following the intervention due to the lack of uniformity in the domains between the different scales. In contrast, it has been possible to investigate the effect of each intervention on the improvement of HL while dichotomously evaluating whether this result variable led to a significant improvement. Trials which obtain a significant improvement of HL provide the best results in improving affective symptomatology. Therefore, the authors suggest that there is a relationship between improving people's HL and improving mental wellbeing. However, it was not possible to measure the baseline symptoms of anxiety and depression of the patients in the trials, meaning that the effect of the interventions was less in patients with diagnosed affective disorders.

As a final limitation, the meta-analysis presents a high degree of heterogeneity and publication bias. This is because the characteristics of the studies have been highly variable and do not present a characteristic homogeneity between the interventions, such as the duration of the intervention and the variety of assessment scales for HL and affective symptomatology. Likewise, there is a tendency for HL interventions to be more directed and structured according to the selected population (patients with chronic conditions, populations, communities, etc.) and, therefore, the diversity of characteristics of literacy interventions that may influence health is higher.

Many HL measurement instruments have been investigated globally and aimed at mental health or specific diseases, as seen in Supplementary Table 4. Some of them, due to their length and complexity, are more typical of the field of research than of clinical practice. The authors of this manuscript, some of whom are GPs that practice healthcare, consider that, in view of the heterogeneity, it would be very useful to generalize the use of short, simple, and applicable scales that would allow clinicians to assess the degree of patients' HL in order to plan the most suitable actions for them. The incorporation of these tools would facilitate the generalization of practices based on lifestyle changes, for which interventions in HL must grant the space required.

Furthermore, the interdisciplinarity of the professionals who apply the intervention may be influential in obtaining better results in mental health; however, this is a limitation of our meta-analysis due to the lack of such information in the collected studies.

## 5. Conclusions

The findings demonstrate that a HL intervention in relation to the symptoms associated with affective disorders improves the emotional state of patients in PHC with a moderately positive effect in reducing depression and anxiety. This effect was greater in individual interventions and more significant in the groups of patients with associated chronic pathologies than in community settings, although an overall improvement was present in all groups.

Generally, a significant improvement in the patients' level of HL also improved their level of mental health by reducing the symptoms associated with anxiety and depression. In patients with diagnosed affective disorders, the effect of the interventions was less. This suggests that prior psychiatric morbidity should be taken into account when programming interventions.

Meanwhile, electronic and individualized HL is increasing. Patients' acceptability and reasons for their preferences must be analyzed and investigated based on sex, age, basic affective disorders, physical limitations, previous level of HL, sociability, and other demographic characteristics. Perhaps in the future, interventions for the recovery of sociability and the improvement of emotional wellbeing should be designed.

E-health tools merit a special mention as they can be strongly incorporated into interventions due to their versatility, flexibility, and ease of implementation by patients. Also, patients' preferences and their reasoning should be taken into consideration and researched further; it is more than likely that the growing individualism of our society favors preferences for tools of an individualistic nature.

There are modulating effects that have not been analyzed in depth throughout this meta-analysis, such as the relationship found in the results between the previous level of education and/or HL and achieving higher scores. A great variability in the interventions was observed, as well as a proper number of scales, some of which focused on specific pathologies and many of which would not be applicable in clinical practice. It is necessary to continue researching the best options to measure and cover all the domains of HL in a uniform way that can be applied to clinical settings and adapted to individual and group situations, population groups, or patients. Interventions should be incorporated into health services to improve the ability of individuals to improve their self-care, level of knowledge of management of their illness, and their physical, mental, and social health. In short, HL is a social and health challenge.

## Data availability statement

The raw data supporting the conclusions of this article will be made available by the authors, without undue reservation.

## Author contributions

LC provided critical feedback on the protocol as part of the project's advisory group. FM-L and DL-I carried out the literature searches. RM-B, FM-L, CB-M, and BO-B contributed to the screening process and the selection of the studies included. FM-L, BO-B, and RM-B extracted the data. CB-M and LC carried out the risk of bias assessments. RM-B, FM-L, and DL-I completed the data analysis. All authors had access to the data, drafted the study protocol, and critically reviewed and approved the manuscript.
