# Supplementary material for: Effectiveness of health literacy interventions on anxious and depressive symptomatology in primary health care: A systematic review and meta-analysis
Source: Front Public Health. 2023 Feb 9;11:1007238. doi: 10.3389/fpubh.2023.1007238 (PMC9948257; doi:10.3389/fpubh.2023.1007238)
Supplement: Supplementary file 1 [file Table_1.pdf]

Supplementary Table 1. Details of search strategy

Database	Initial	Last 10 years	Only RCTs
Medline	501	443	49
Embase	268	212	23
Web Of Science	1,344	1,106	123
Ibecs	26	8	2
Cuiden	3	3	3
Scielo	404	238	5
Science Direct	277	242	137
Dialnet	16	13	8
Grey Literature	24	24	0
**Total**	2,863	2,289	350

**MEDLINE** 2011 to 2022 Only RCTs		*studies*
1	health literacy[MeSH Terms] OR health literacy[Title/Abstract] OR health knowledge[Title/Abstract]	787
2	depressive disorder[MeSH Terms] OR depressive disorder[Title/Abstract] OR depression[MeSH Terms] OR depression[Title/Abstract] OR depressive*[Title/Abstract]	15,792
3	Anxiety Disorders[MeSH Terms] OR Anxiety Disorders[Title/Abstract] OR Anxiety[MeSH Terms]))] OR Anxiety[Title/Abstract] OR	11,334
4	"Mood Disorders"[MeSH Terms] OR "Mood Disorders"[Title/Abstract] OR "Affective Disorder"[Title/Abstract]	4,502
5	adult[MeSH Terms] OR adult[Title/Abstract] OR adult*[Title/Abstract] OR aged[MeSH Terms] OR aged[Title/Abstract] OR aged*[Title/Abstract]	207,818
6	((((health literacy[MeSH Terms]) OR (health literacy[Title/Abstract]) OR (health knowledge[Title/Abstract])) AND (((((depressive disorder[MeSH Terms]) OR (depression[MeSH Terms]) OR (depressive disorder[Title/Abstract]) OR (depression[Title/Abstract]) OR (depressive*[Title/Abstract]) OR (Anxiety Disorders[MeSH Terms]) OR (Anxiety[MeSH Terms])) OR (Anxiety Disorders[Title/Abstract])) OR (Anxiety[Title/Abstract]) OR ("Mood Disorders"[MeSH Terms] OR "Mood Disorders"[Title/Abstract] OR "Affective Disorder"[Title/Abstract]))) AND (((((adult[MeSH Terms]) OR (aged[MeSH Terms]) OR (adult[Title/Abstract]) OR (aged[Title/Abstract]))) OR (aged*[Title/Abstract])) OR (adult*[Title/Abstract]))	49

**EMBASE** 2011 to 2022 Only RCTs		*studies*
1	'health literacy':ti,ab,kw OR 'attitude to health':ti,ab,kw	855
2	'depression':ti,ab,kw	22,205
3	'anxiety disorder':ti,ab,kw OR 'anxiety':ti,ab,kw	16,500
4	'mood disorder':ti,ab,kw	201
5	adult:ti,ab,kw OR aged:ti,ab,kw	60,595
6	(('depression':ti,ab,kw OR 'anxiety disorder':ti,ab,kw OR 'anxiety':ti,ab,kw OR 'mood disorder':ti,ab,kw) AND ('health literacy':ti,ab,kw OR 'attitude to health':ti,ab,kw) AND (adult:ti,ab,kw OR aged:ti,ab,kw))	23

**WEB OF SCIENCE** 2011 to 2022		*studies*
1	TS= health-literacy OR TI= health-literacy OR AB= health-literacy OR AK= health-literacy OR KP= health-literacy	14,780
2	TS= depression OR TI= depression OR AB= depression OR AK= depression OR KP= depression	360,073
3	TS= anxiety-disorder OR TI= anxiety-disorder OR AB= anxiety-disorder OR AK= anxiety-disorder OR KP= anxiety-disorder	36,620
4	TS= mood-disorder OR TI= mood-disorder OR AB= mood-disorder OR AK= mood-disorder OR KP= mood-disorder OR TS= affective-disorder OR TI= affective-disorder OR AB= affective-disorder OR AK= affective-disorder OR KP= affective-disorder	25,898
5	(TS= (adult OR aged) OR TI= (adult OR aged) OR AB= (adult OR aged) OR AK= (adult OR aged) OR KP= (adult OR aged))	3,033,090
6	(TS= health-literacy OR TI= health-literacy OR AB= health-literacy OR AK= health-literacy OR KP= health-literacy) AND (TS= depression OR TI= depression OR AB= depression OR AK= depression OR KP= depression OR TS= mood-disorder OR TI= mood-disorder OR AB= mood-disorder OR AK= mood-disorder OR KP= mood-disorder OR TS= affective-disorder OR TI= affective-disorder OR AB= affective-disorder OR AK= affective-disorder OR KP= affective-disorder OR TS= anxiety-disorder OR TI= anxiety-disorder OR AB= anxiety-disorder OR AK= anxiety-disorder OR KP= anxiety-disorder) AND (TS= (adult OR aged) OR TI= (adult OR aged) OR AB= (adult OR aged) OR AK= (adult OR aged) OR KP= (adult OR aged))	1,106

**IBECS** No date limit		*studies*
1	"ALFABETIZACIÓN" [Palabras] and "SALUD" [Palabras]	399
2	"DEPRESIÓN" [Palabras]	5,899
3	"ANSIEDAD" [Palabras]	3,729
4	"DEPRESIÓN" [Palabras] and "ALFABETIZACIÓN" [Palabras] and "SALUD" [Palabras]	14
5	"ANSIEDAD" [Palabras] and "ALFABETIZACIÓN" [Palabras] and "SALUD" [Palabras]	12

**CUIDEN** No date limit		*studies*
1	[cla="Alfabetización en Salud"]	65
2	[cla="Depresión"]	1,797
3	[cla="Ansiedad"]	2,531
4	[cla="Alfabetización en Salud"] and [cla="Depresión"]	2
5	[cla="Alfabetización en Salud"] and [cla="Ansiedad"]	1

**SCIELO** 2011 to 2022		*studies*
1	(ti:(health literacy)) OR (ab:(health literacy)) OR (kw:(health literacy))	561
2	(ab:(depression)) OR (kw:(depression)) OR (ti:(depression))	6,996
3	(ab:(anxiety)) OR (kw:(anxiety)) OR (ti:(anxiety))	5,648
4	(ab:(mood disorder)) OR (kw:(mood disorder)) OR (ti:(mood disorder))	290
5	(ab:(anxiety)) OR (ti:(depression)) OR (kw:(depression)) OR (kw:(anxiety)) OR (kw:(mood disorder)) OR (ti:(anxiety)) OR (ti:(mood disorder)) OR (ab:(depression)) OR (ab:(mood disorder)) AND (ti:(health literacy)) OR (ab:(health literacy)) OR (kw:(health literacy))	238

**SCIENCE DIRECT** 2011 to 2022		*studies*
1	(health literacy OR alfabetizacion en salud)	41,059
2	depressive disorder OR depression OR depresión OR trastorno depresivo	483,646
3	anxiety disorder OR anxiety OR ansiedad OR trastorno ansiedad	312,791
4	mood disorder OR affective OR afectivo OR trastorno afectivo	2,935,155
5	adult OR aged OR adulto	3,088,031
6	(depressive disorder OR depression OR anxiety disorder OR anxiety OR ansiedad OR mood disorder OR affective) AND (health literacy OR alfabetizacion en salud)	242

**DIALNET** 2011 to 2022		*studies*
1	health literacy	535
2	Depresión	7,639
3	Ansiedad	8,076
4	Depresión AND Ansiedad AND health literacy	13
