# Supplementary material for: Effectiveness of health literacy interventions on anxious and depressive symptomatology in primary health care: A systematic review and meta-analysis
Source: Front Public Health. 2023 Feb 9;11:1007238. doi: 10.3389/fpubh.2023.1007238 (PMC9948257; doi:10.3389/fpubh.2023.1007238)
Supplement: Supplementary file 2 [file Table_2.pdf]

frontiers

Supplementary Table 2: GRADE summary of findings and certainty of evidence for each outcome domain

Health literacy interventions on anxious and depressive symptomatology in Primary Health Care

Population/setting: Adult population of Primary Health Care.
Intervention: Health literacy interventions
Comparison: Usual Care in Primary Health Care
Outcome: Improvement of anxious and depressive symptomatology

Outcomes	Risk with no intervention	Risk with intervention [95% CI]	Effect estimate [95% CI]	No. of participants (studies)	Duration intervention (weeks)	Certainty of the evidence (GRADE)	Comments
**1. Improvement of depressive symptomatology.**							
**1.1. Digital systems HL interventions decrease depression scores.**							
			EFFECT SIZE				
a) ↓ PHQ-9 scores (Range 1-27)	Mean PHQ-9 score 8.14	Mean **2.33 pts lower** [-2.59, -2.06]	SMD -1.891 [-2.319, -1.463]	397 (2 studies)	Mean 16 weeks (Range 4-52)	**Moderate** ⊕⊕⊕⊖	- Downgraded for serious inconsistency as heterogeneity (I2 >75% in meta-analysis)<br>- Downgraded for suspected publication bias given impression from funnel plot<br>- Upgrade for suspected positive influence of Residual Confounding
b) ↓ BDI-II scores (Range 0-63)	Mean BDI-II score 8.48	Mean **0.84 pts lower** [-1.04, -0.65]	SMD -0.028 [-0.304, 0.249]	202 (1 studies)	2 weeks	**Low** ⊕⊕⊖⊖	- Downgraded for serious inconsistency as heterogeneity (I2 >75% in meta-analysis)<br>- Downgraded for RoB2 some concerns for bias<br>- Downgraded for imprecision<br>- Downgraded for suspected publication bias given impression from funnel plot<br>- Downgraded for suspected negative influence of Residual Confounding
**1.2. HL group interventions decrease depression scores.**							
			EFFECT SIZE				
a) ↓ PHQ-9 scores (Range 1-27)	Mean PHQ-9 score 12.16	Mean **2.34 pts lower** [-3.61, -1.06]	SMD -0.235 [-0.547, 0.077]	241 (1 studies)	10 weeks	**Low** ⊕⊕⊖⊖	- Downgraded for imprecision and indirectness<br>- Downgraded for suspected publication bias given impression from funnel plot<br>- Downgraded for suspected negative influence of Residual Confounding
b) ↓ GSD-5 scores (Range 0-5)	Mean GSD-5 score 3.15	Mean **1.18 pts lower** [-1.74, -0.62]	SMD -0.499 [-0.865, -0.134]	418 (2 studies)	Mean 18 weeks (Range 12-24)	**Moderate** ⊕⊕⊕⊖	- Downgraded for indirectness<br>- Downgraded for suspected publication bias given impression from funnel plot<br>- Downgraded for suspected negative influence of Residual Confounding
**1.3. HL individual face to face interventions decrease depression scores.**							
			EFFECT SIZE				
a) ↓ PHQ-9 scores (Range 1-27)	Mean PHQ-9 score 14.3	Mean **7.30 pts lower** [-8.42, -6.17]	SMD -0.571 [-0.892, -0.251]	228 (1 studies)	24 weeks	**High** ⊕⊕⊕⊕	- Downgraded for RoB2 some concerns for bias<br>- Downgraded for imprecision<br>- Upgrade for very large effect

**1.4. HL individual telephonic interventions decrease depression scores.**							
EFFECT SIZE							
a) ↓ PHQ-9 scores (Range 1-27)	Mean PHQ-9 score 16.9	Mean **3.80 pts lower** [-3.86, -3.74]	SMD -1.898 [-2.104, -1.692]	609 (1 studies)	16 weeks	**High** ⊕⊕⊕⊕	- Downgraded for serious inconsistency as heterogeneity (I2 >75% in meta-analysis)<br>- Downgraded for suspected publication bias given impression from funnel plot<br>- Upgrade for large effect
b) ↓ CES-D scores (Range 0-60)	Mean CES-D score 12.35	Mean **0.93 pts lower** [-1.11, -0.75]	SMD -0.512 [-0.512, -0.194]	216 (1 studies)	4 weeks	**Low** ⊕⊕⊖⊖	- Downgraded for serious inconsistency as heterogeneity (I2 >75% in meta-analysis)<br>- Downgraded for indirectness<br>- Downgraded for suspected publication bias given impression from funnel plot
**2. Improvement of anxious symptomatology.**							
**2.1. Digital systems HL interventions decrease anxiety scores.**							
EFFECT SIZE							
a) ↓ GAD - 7 score (Range 1-21).	Mean GAD-7 score 6.09	Mean **1.86 pts lower** [-2.09, -1.64]	SMD. -2.318 [-2.786, -1.851]	397 (2 studies)	Mean 16 weeks (Range 4-52)	**Moderate** ⊕⊕⊕⊖	- Downgraded for serious inconsistency as heterogeneity (I2 >75% in meta-analysis)<br>- Downgraded for suspected publication bias given impression from funnel plot<br>- Upgrade for suspected positive influence of Residual Confounding
**2.2. HL individual telephonic decrease of anxiety scores.**							
EFFECT SIZE							
b) ↓ GAD - 7 score (Range 1-21).	Mean GAD-7 score 12.95	Mean **4.80 pts lower** [-4.85, -4.74]	SMD. -3.463 [-3.763, -3.191]	609 (1 studies)	16 weeks	**High** ⊕⊕⊕⊕	- Downgraded for serious inconsistency as heterogeneity (I2 >75% in meta-analysis)<br>- Downgraded for suspected publication bias given impression from funnel plot<br>- Upgrade for large effect

SMD: Standardised Mean Difference; 95% CI: 95% Confidence Interval; PHQ: Patient Health Questionnaire; GAD-7: General Anxiety Disorder; GDS: Geriatric Depression Scale; CES-D: Center for Epidemiological Studies Depression Scale; BDI-II: Beck Depression Inventory; I2: I2 test Higgins y Thompson; Rob2: Cochrane Collaboration Revised Risk of Bias tool

**GRADE Working Group grades of evidence**	
**High certainty** ⊕⊕⊕⊕	We are very confident that the true effect lies close to that of the estimate of the effect
**Moderate certainty** ⊕⊕⊕⊖	We are moderately confident in the effect estimate: The true effect is likely to be close to the estimate of the effect, but there is a possibility that it is substantially different
**Low certainty** ⊕⊕⊖⊖	Our confidence in the effect estimate is limited: The true effect may be substantially different from the estimate of the effect
**Very low certainty** ⊕⊖⊖⊖	We have very little confidence in the effect estimate: The true effect is likely to be substantially different from the estimate of effect
