# Supplementary material for: Effectiveness of health literacy interventions on anxious and depressive symptomatology in primary health care: A systematic review and meta-analysis
Source: Front Public Health. 2023 Feb 9;11:1007238. doi: 10.3389/fpubh.2023.1007238 (PMC9948257; doi:10.3389/fpubh.2023.1007238)
Supplement: Supplementary file 3 [file Table_3.pdf]

frontiers

Supplementary Table 3: Risk of bias assessment domains detailed for each study.

Bias domain and signalling question*	Bakker D et al. 2018	Blancafort-Alias S et al. 2021	Bohingam U et al. 2018	Heckel et al. 2018	Johnson J et al 2015	Kiropoulos L et al. 2011	Salisbury C et al. 2016	Uemura K et al. 2021	Van-Dyke BP et al. 2019
**1. Bias arising from the randomization process**									
1.1. Was the design sequence random?	Y	Y	Y	Y	Y	Y	Y	Y	Y
1.2. Was the allocation sequence concealed until participants were included and allocated to interventions?	N	Y	Y	Y	N	N	Y	Y	Y
1.3. Did baseline differences between intervention groups suggest a problem with the randomisation process?	N	N	N	N	N	N	N	N	N
Bias risk judgment (low/high/some concerns)	?	+	+	+	?	+	+	+	+
**2. Bias due to deviations from intended interventions**									
2.1. Were participants aware of their assigned intervention during the trial?	N	Y	Y	Y	N	N	Y	Y	Y
2.2. Were caregivers and people delivering the interventions aware of the participant-focused intervention during the trial?	N	Y	Y	Y	N	N	Y	Y	Y
2.3. If Y/PY/NI to 2.1 or 2.2: Were there deviations from the intended intervention that arose due to the trial setting?	NA	N	N	N	NA	NA	N	N	N
2.4. If Y/PY/NI to 2.3: Are these deviations likely to have affected the result?	NA	NA	NA	NA	NA	NA	NA	NA	NA
2.5. If S/PY to 2.4: Were these deviations from the intended intervention balanced btw groups?	NA	NA	NA	NA	NA	NA	NA	NA	NA
2.6. Was an appropriate analysis used to estimate the effect of intervention allocation?	Y	Y	Y	Y	Y	Y	Y	Y	Y
2.7. If N/PN/NI to 2.6: Was there the potential for a substantial impact (on the outcome) of the lack of analysis of the participants in the group to which they were randomised?	NA	NA	NA	NA	NA	NA	NA	NA	NA
Bias risk judgment (low/high/some concerns)	?	+	+	+	?	+	+	+	+
**3. Bias due to missing outcome data**									
3.1. Were data for this outcome available for all, or almost all, randomized participants?	Y	Y	Y	Y	Y	Y	Y	Y	Y
3.2. If N/PN/NI to 3.1: Is there evidence the result was not biased by missing outcome data?	NA	NA	NA	NA	NA	NA	NA	NA	NA
3.3. If N/PN to 3.2: Could the absence in the result depend on its true value?	NA	NA	NA	NA	NA	NA	NA	NA	NA
3.4. If S/PY/NI to 3.3: Is the omission in the result likely to depend on its actual value?	NA	NA	NA	NA	NA	NA	NA	NA	NA
Bias risk judgment (low/high/some concerns)	+	+	+	+	+	+	+	+	+
**4. Bias in measurement of the outcome**									
4.1. Was the outcome measurement method inappropriate?	N	N	N	N	N	N	N	N	N
4.2. Could the measurement or determination of the outcome have been different between the intervention groups?	Y	Y	Y	Y	Y	Y	Y	Y	Y
4.3. If N/PN/NI to 4.1 and 4.2: Were the outcome assessors aware of the intervention that the study participants received?	Y	Y	Y	Y	Y	Y	Y	Y	Y
4.4. If Y/PY/NI to 4.3: Could the outcome assessment have been influenced by knowledge of the intervention received?	Y	N	N	N	Y	N	N	N	N
4.5. If Y/PY/NI to 4.4: Is it likely that the outcome assessment was influenced by knowledge of the intervention received?	Y				Y				
Bias risk judgment (low/high/some concerns)	-	?	?	?	-	?	?	?	?
**5. Bias in selection of the reported result**									
5.1. Were the data producing this outcome analysed according to a pre-specified analysis plan that was finalized before unblinded outcome data were available for analysis?	Y	Y	Y	Y	Y	Y	Y	Y	Y
5.2. Is it likely that the numerical result being evaluated has been selected, on the basis of the results, from multiple eligible outcome measures (eg, scales, definitions, time points) within the outcome domain?	Y	N	N	Y	N	Y	N	Y	N
5.3. Is it likely that the numerical result being evaluated has been selected, on the basis of the results, from multiple eligible analyses of the data?	Y	Y	Y	Y	Y	Y	Y	Y	Y
Bias risk judgment (low/high/some concerns)	+	?	?	+	?	?	?	+	?
**6. Overall bias**									
Bias risk judgment (low/high/some concerns)	?	+	+	+	?	?	+	+	+

Y:yes; PY: probably yes; PN: probably not; N:no; NA: not applicable; NI: no information; +:Low Risk; ?: some concerns; -:High Risk
