# Supplementary material for: Effectiveness of health literacy interventions on anxious and depressive symptomatology in primary health care: A systematic review and meta-analysis
Source: Front Public Health. 2023 Feb 9;11:1007238. doi: 10.3389/fpubh.2023.1007238 (PMC9948257; doi:10.3389/fpubh.2023.1007238)
Supplement: Supplementary file 4 [file Table_4.pdf]

Supplementary Table 4: Questionnaires employed.

Depressive symptomatology

Patient health questionnaire (PHQ-9)

The patient health questionnaire (PHQ) is a 3-page self-administered version of the PRIME-MD. At 9 items, the PHQ depression scale (which we call the PHQ-9) is thus a dual-purpose instrument that, with the same nine items, can establish provisional depressive disorder diagnoses as well as grade depressive symptom severity. The internal reliability of the PHQ-9 was excellent, with a Cronbach's α value of 0.89.
The standard cut points are

- 1 to 4 None
- 5 to 9 Mild
- 10 to 14 Moderate
- 15 to 19 Moderately Severe
- 20 to 27 Severe

Geriatric Depression Scale (GSD-5)

Self-report assessment is used to identify depression in the elderly. This is a short version with 5 items.
The authors found that a score of 5 or more positive responses suggests depression and found a sensitivity of 97% and specificity of 85% on the GDS-5.
The scored answers are affirmative in items 2, 3, 4 and 5, and negative in item 1.
Each correct answer scored 1.
A score of 2 or more indicates depression

Center for Epidemiologic Studies Depression Scale (CES-D)

Self-report scale of 20 items that measures the frequency of feelings and behaviour during the last seven days, scoring each response from 0 to 3, obtaining a score range from 0 to 60.
Higher scores indicated a greater frequency of depressive experiences.
This scale has high internal consistency in community and clinical populations (Cronbach's αs .85−.90).

Beck Depression Inventory-II (BDI-II)

This is a self-report inventory to measure the severity of depression, consisting of 21 multiple-choice questions, scoring each response on a scale of 0 to 3. It was translated and validated into Spanish with a reliability of 0.89.
Standardised cut-off points are

- 0 to 13 Minimal /no depression
- 14 to 19 Mild
- 20 to 28 Moderate
- 29 to 63 Severe

Supplementary Table 4: Questionnaires employed (continued)

Anxiety symptomatology

**Generalized Anxiety Disorder Scale GAD-7**
This is a self-administered scale consisting of 7 questions scored between 0 and 3, with the minimum and maximum possible scores being 0 and 21, respectively.<br>The standard cut points are<br>- 1 to 4 Anxiety is not appreciated<br>- 5 to 9 Mild<br>- 10 to 14 Moderate<br>- 15 to 21 Severe

Health Literacy

**Mental Health Literacy Questionnaire (MHLQ-25)**
The MHLq is a practical, valid, and reliable tool to identify gaps in knowledge, beliefs and behavioural intentions, in large samples, which allows the development and evaluation of interventions aimed at promoting mental health.<br>The questionnaire showed good internal consistency (total score α=0.84) and excellent test-retest reliability, the ICC for the total MHLq score was 0.88.

**European Health Literacy Survey (HLS-EU-Q16)**
The HLS-EU-Q16 in Spanish is a brief, adequate and valid instrument to measure the level of health literacy of the population. The HLS-EU-Q16 questionnaire consists of 16 questions, with dichotomous response options: very difficult and difficult=0, easy and very easy=1<br>Reliability was high (intraclass correlation coefficient: 0.923; kappa: 0.814). Factor analysis suggested a unifactorial structure (79.1% of variability explained by the common factor), with high factor loadings. The consistency was high (Cronbach's alpha: 0.982). The domains in which this questionnaire is divided are<br>- Healthcare: The ability to access information on medical or clinical issues, to understand and evaluate medical information, and to make informed decisions on medical issues and comply with medical advice.<br>- Disease prevention: The ability to access information on risk factors for health, to understand and evaluate information about risk factors, and to make informed decisions to protect against risk factors for health.<br>- Health promotion: The ability to regularly update oneself on determinants of health in the social and physical environment and derive meaning, to interpret and evaluate information on determinants of health in the social and physical environment.<br>The standard cut points are<br>- 0 to 12 inadequate or problematic level.<br>- 13 to 16 level enough.

Supplementary Table 4: Questionnaires employed (continued)

Health Education Impact Questionnaire (heiQ)

User-friendly, relevant, and psychometrically sound instrument for the comprehensive evaluation of patient education programmes, which can be applied across a broad range of chronic conditions. This questionnaire has 42 items with likert type question and eight dimensions, The heiQ has high construct validity and is a reliable measure of a broad range of patient education programme benefits (high Cronbach's alpha: 0.70–0.89)
Eight independent dimensions were derived:
- Positive and active engagement in life: High scores characterise people who are actively engaged in life and are motivated to improve their life circumstances
- Health-directed behaviour: High scores indicate high levels of healthful behaviour including prevention, diet, and exercise
- Self-monitoring and insight: High scores identify self-monitoring, self-management, setting reasonable limits or targets, and insight into living with a health problem
- Constructive attitudes and approaches: High scores identify individuals who attempt to minimise the effects of illness and are determined not to allow the illness to control their life
- Skill and technique acquisition: High scores are characteristic of someone who has highly developed skills in symptom relief and techniques to manage own health
- Social integration and support: High scores indicate high levels of social interaction, high sense of support, seeking support from others, and low levels of feelings of social isolation due to illness
- Health service navigation: High scores characterise a person who is confident in their ability to communicate with healthcare professionals and have a good understanding of ways to access healthcare to get their needs met.
- Emotional Wellbeing: High scores identify individuals who have high levels of overall health-related negative affect, negative attitude towards life, and high levels of anxiety, stress, anger, and depression

3 Health Literacy Questions (3HLQ)
The previously validated set of 3 brief of questions that screened for inadequate Health Literacy (HL)
The questions were
- How often do you have problems learning about your medical condition because of difficulty understanding written information?
- How confident are you filling out medical forms by yourself?
- How often do you have someone like a family member, friend, hospital or clinic worker or caregiver help you read health plan materials (such as written information about your health or care you are offered)?
These questions were scored on a 5-point Likert scale, with higher scores indicating lower HL. We used a weighted summative score of the 3 HL items and stratified summative scores such that:
- 0 to 9 Adequate HL
- 9 15 Inadequate HL
The Cronbach's alpha coefficient was 0.53 for the 3-SQ.

Depression literacy Scale (D-Lit scale)
Depression literacy was assessed using translated and adapted versions of the D-Lit scale that is a 22-item test of knowledge about depression with dichotomous response options: false=0, true=1.
The internal reliability analysis of this sample indicated an alpha coefficient of 0.88 - 0.92
A higher score on this scale indicated greater literacy.

Supplementary Table 4: Questionnaires employed (continued)

eHEALs literacy scale (eHEALs)
This is a self-administered measure of eHealth literacy developed to measure consumers' combined knowledge, comfort, and perceived skills at finding, evaluating, and applying electronic health information to health problems. This is a questionnaire with 8 items where higher scores on this scale indicated greater literacy.
The Cronbach's alpha coefficient was 0.88

Health Literacy Scale (HLS-14)
This is a scaled with 14 items and 3 dimensions:
- Functional Health Literacy: To assess the extent to which individuals possess the basic abilities required to read instructions or leaflets that have been provided by hospitals or pharmacies.
- Communicative Health Literacy: To assess the extent to which individuals possess the more advanced skills that are necessary to obtain information about diseases and treatments as well as to use this information in everyday life.
- Critical Health Literacy: To assess the extent to which individuals possess the more advanced skills that are necessary to critically analyze information about diseases and treatments and use this information to exert greater control over life events and situations.

The questions were scored on a 5-point Likert scale
Higher scores indicate higher health literacy.
The Cronbach's alpha coefficient was 0.83, 0.85 and 0.76 in each dimension respectively

Short Form of the Test of Functional Health Literacy in Adults (S-TOFHLA)
This scale measures patients' ability to read and apply health-related information that is commonly encountered in medical settings.
To complete the test participants, must read 2 passages of text with omitted words and select the correct response from a list of options.
Score range:
- 0 to 16 In adequate functional health literacy
- 17 to 22 Marginal functional health literacy
- 23 to 36 Adequate functional health literacy

The S-TOFHLA has an excellent internal consistency (α = .97) for reading comprehension and a strong correlation (r = .80)
