# Supplementary material for: Effectiveness of health literacy interventions on anxious and depressive symptomatology in primary health care: A systematic review and meta-analysis
Source: Front Public Health. 2023 Feb 9;11:1007238. doi: 10.3389/fpubh.2023.1007238 (PMC9948257; doi:10.3389/fpubh.2023.1007238)
Supplement: Supplementary file 5 [file Table_5.pdf]

Supplementary Table 5: Health literacy outcomes

Author. Year	Sample size	Health Literacy Scale	Baseline		Post-Intervention	
			Intervention Mean ± SD; % (n)	Control Mean ± SD; % (n)	Intervention Mean ± SD; % [CI95%]	Control Mean ± SD; % [CI95%]
Bakker D et al. 2018	N= 226<br>IG1= 56<br>IG2= 56<br>IG3= 50<br>CG= 64	**MHLQ-25**	IG1= 16.40 ± 2.09<br>IG2= 16.54 ± 2.42<br>IG3= 16.20 ± 2.44	16.38 ± 2.12	IG1= 16.26 ± 2.34<br>IG2= 16.57 ± 1.83<br>IG3= 16.28 ± 2.6	16.61 ± 2.22
Blancafort-Alias S et al. 2021	N= 358<br>IG= 194<br>CG=164	**HLS-EU**				
		Understand medical information	25.51 (50)	15.85 (26)	-0.62 [-1.10 to -0.13]*	-0.45[-0.99 to 0.08]
		Find out about mental health activities	32.65 (64)	25.61 (42)	-0.45[-0.92 to 0.02]	0.34 [-1.17 to 0.85]
		Assess healthy lifestyles	17.35 (34)	14.03 (23)	-0.24[-0.72 to 0.23]	-0.00 [-0.51 to 0.51]
Bohingamu S et al. 2018	N = 171<br>IG = 86<br>CG = 85	**HeiQ**				
		Health-directed behaviour			0.09 ± 0.49	-0.08 ± 0.69
		Positive and active engagement in life			0.11 ± 0.59*	-0.16 ± 0.55
		Self-monitoring and insight			0.13 ± 0.44*	-0.08 ± 0.43
		Constructive attitudes and approaches			0.12 ± 0.45	-0.01 ± 0.48
		Skill and technique acquisition			0.17 ± 0.52*	-0.05 ± 0.50
		Social integration and support			0.12 ± 0.52*	-0.19 ± 0.63
		Health services navigation			-0.10 ± 0.75	0.02 ± 0.57
		Emotional distress			0.04 ± 0.45	0.00 ± 0.55
Heckel L et al. 2018	N = 216<br>CG= 108<br>IG= 108	**HeiQ**				
		Health-directed behaviour	2.83 ± 0.07	2.90 ± 0.07	2.81 ± 0.08	3.05 ± 0.08
		Positive and active engagement in life	3.05 ± 0.05	3.15 ± 0.05	3.02 ± 0.05	3.18 ± 0.06
		Self-monitoring and insight	1.78 ± 0.06	1.70 ± 0.06	1.95 ± 0.06	1.73 ± 0.07
		Constructive attitudes and approaches	3.16 ± 0.04	3.17 ± 0.04	3.07 ± 0.04	3.13 ± 0.05
		Skill and technique acquisition	3.31 ± 0.05	3.38 ± 0.05	3.20 ± 0.05	3.30 ± 0.05
		Social integration and support	3.15 ± 0.04	3.17 ± 0.04	3.01 ± 0.05	3.04 ± 0.05
		Health services navigation	3.12 ± 0.05	3.23 ± 0.05	2.98 ± 0.05	3.08 ± 0.05
		Emotional distress	3.17 ± 0.05	3.27 ± 0.05	3.07 ± 0.05	3.23 ± 0.05
Johnson J et al. 2015	N = 228<br>IG = 95<br>CG1 = 71<br>CG2 = 62	**3HLQ**	HL > 9 = 16%		5.9 ± 2.6	
Kiropoulos L et al. 2011	N = 202<br>IG = 110<br>CG = 92	**Depression literacy**	10.61 ± 3.28	8.17 ± 4.29	17.43 ± 3.99 *	8.03 ± 4.33
Salisbury C et al. 2016	N = 609<br>CG = 302<br>IG = 307	**Health literacy (eHEALs)**	3.7 ± 0.8	3.6 ± 0.9	3.9 ± 0.8*	3.7 ± 0.8
		**HeiQ**				
		Health-directed behaviour	2.4 ± 0.9	2.4 ± 0.9	3.0 ± 0.5*	2.4 ± 0.9
		Positive and active engagement in life				
		Self-monitoring and insight	2.9 ± 0.4	2.8 ± 0.4	2.9 ± 0.4	2.6 ± 0.6
		Constructive attitudes and approaches	2.6 ± 0.6	2.5 ± 0.6	2.8 ± 0.5	2.6 ± 0.5
		Skill and technique acquisition	2.6 ± 0.5	2.6 ± 0.5	2.9 ± 0.6*	2.8 ± 0.6
		Social integration and support				
		Health services navigation	2.8 ± 0.6	2.7 ± 0.6	3.2 ± 1.1*	3.4 ± 0.9
		Emotional distress				
Uemura K et al. 2021	N = 60<br>CG = 30<br>IG = 30	Health Literacy Scale-14 scores				
		Functional health literacy	19.6 ± 4.0	19.2 ± 4.3	20.3 ± 4.7	20.8 ± 5.3
		Communicative health literacy	17.3 ± 4.2	17.4 ± 3.5	19.5 ± 5.2*	17.3 ± 3.6
		Critical health literacy	13.9 ± 3.5	13.5 ± 3.5	15.2 ± 4.0	13.4 ± 3.5
Van-Dyke BP et al. 2019	N = 241<br>CG = 78<br>IGCBT=83<br>IGEDU=80	STOFHLA	CBT:27.36 ± 9.09<br>EDU: 32.25 ± 7.06	32.45 ± 4.84	CBT:27.41 ± 9.1	32.39 ± 4.9

N: Total Sample; CI 95%: Confidence Interval 95 %; CG: Control Group; IG: Intervention Group; SD: Standard Deviation; IG1: Intervention Group 1(Moodkit); IG2: Intervention Group 1(Moodprism); IG2: Intervention Group3 (Moodmission); CG1: Control Group 1 (active control); CG2: Control Group 2 (usual care);HL:health literacy; CBT: Cognitive Behavioural treatment; IGCBT: Literacy-adapted group CBT; IGEDU: psychoeducation groups; MHLQ; Mental Health Literacy Questionnaire; HLS-EU: European Health Literacy Survey; heiQ: Health Education Impact Questionnaire; HLQ: Health Literacy Questionnaire; 3QHL: 3 Question of Health Literacy; D-Lit: Depression Literacy Questionnaire; eHEALs: eHealth literacy scale; S-TOFHLA: Abbreviated version of the Test of Functional Health Literacy in Adults.
